# Complications after intramedullary nail fixation of pathological versus non-pathological femoral shaft fractures: a retrospective cohort study in 233 patients

**DOI:** 10.1186/s13037-021-00304-7

**Published:** 2021-08-26

**Authors:** Chirathit Anusitviwat, Khanin Iamthanaporn, Pakjai Tuntarattanapong, Boonsin Tangtrakulwanich, Tippawan Liabsuetrakul

**Affiliations:** 1 Department of Orthopedics, Faculty of Medicine, Prince of Songkla University, 15 Karnjanavanich Road, 90110 Hat Yai, Songkhla Thailand; 2 Department of Epidemiology, Faculty of Medicine, Prince of Songkla University, Hat Yai, Songkhla Thailand

**Keywords:** Pathological fracture, Femoral shaft, Metastasis, Intramedullary nail

## Abstract

**Background:**

Postoperative adverse events after intramedullary nailing have been reported in patients with metastatic pathological and non-pathological femoral fractures. Other consequences to be considered are readmission and reoperation. Few studies have compared the risks of postoperative adverse events, reoperation, and readmission after intramedullary nailing of pathological and non-pathological femur fractures. This study was designed to test the hypothesis that patients with pathological femoral fractures had more adverse events, readmission, and reoperation following surgical fixation than non-pathological femoral fractures.

**Methods:**

This was a retrospective observational cohort study, conducted at an academic medical center in Thailand. The data from patients with femoral shaft fractures undergoing long intramedullary nailing, from June 1, 2006, to June 30, 2020, were included. Patients who had a pathological fracture from a primary bone tumor, metabolic bone disease, or inadequate/missing information were excluded. Patients with pathological fractures from metastatic bone disease were assigned to be the pathological group whereas those with traumatic fractures were assigned to be the non-pathological group. The primary outcome was the risk of inpatient adverse events as compared between the two groups. The secondary outcome was the risk of consequences after discharge as compared between the two groups. Outcomes were analyzed by using multivariate logistic regression analysis.

**Results:**

The total number of patients was 48 in the pathological fracture group and 185 in the non-pathological group. There were significantly higher rates of surgical and medical adverse events in patients with pathological fractures compared to patients with non-pathological fractures. After adjusting for potential confounding factors in multivariate regression analysis, patients with pathological fractures had higher odds of both adverse surgical (adjusted OR 2.43, 95 % CI 1.15–5.13) and medical adverse events (adjusted OR 2.81, 95 % CI 1.13–7.03).

**Conclusions:**

Patients with metastatic pathological femoral shaft fractures undergoing intramedullary nailing were more likely to experience postoperative adverse events than patients with non-pathological fractures.

## Introduction

Femoral shaft fractures are common trauma injuries with high global incidence rates [1]. These fractures usually result from either high or low energy injuries; the latter frequently occur in patients with bones weakened through some pathology [2–4]. Pathological femoral fractures can result from various underlying diseases, such as infection, metabolic bone diseases, or bone tumors. Among bone tumors, metastatic disease is the most frequent malignant neoplasm of the bone, ranging from 25 to 85 %, and usually originating from the prostate, breast, lung, kidney, or thyroid [5–8]. The femur is the second most common metastasis site following the vertebra [9, 10], with high susceptibility of progression to pathological fractures because the femur is a long, high load-bearing bone [11, 12]. Pathological fractures of the femur are associated with severe pain, immobilization, and diminished quality of life [7, 13]. Therefore, these patients require prompt surgical intervention to restore their function and relief pain [14–17].

Surgical fixation of metastatic bone disease is principally indicated for pathological femoral fractures [14, 16]. Durable implants and mechanically stable internal fixation need to be considered due to the poor bony union of pathological fractures [11, 18]. Antegrade intramedullary nailing is an option for treating pathological femoral shaft fractures, since several studies have reported that patients treated with this method had good functional outcomes [19–23]. Although the benefits of internal fixation after a pathological fracture of the femur are known, patients who incurred adverse events following this surgical procedure have been reported [14, 24–27].

Postoperative adverse events reported in patients with femoral fractures showed relatively higher rates of adverse events in pathological fractures than those in non-pathological fractures; even non-pathological fractures have been associated with severe soft tissue injuries and multiple surgical procedures [7, 15, 28]. However, these studies included patients with different characteristics and various surgical procedures, for which the adverse events were not comparable. These adverse events have been reported to increase mortality in patients with pathological fractures after an operation [7, 29]. Few studies have assessed the risk of reoperation and readmission, which are essential for further treatment planning [27]. Hence, this study we analyzed not only adverse events, but also their consequences, including readmission and reoperation. This study aimed to compare the risk of inpatient adverse events and complications after discharge between patients with metastatic pathological and non-pathological femoral fractures undergoing intramedullary nailing. It was hypothesized that patients with pathological femoral fractures had more adverse events, reoperations, and readmissions following surgical fixation.

## Methods

### Study design and sample

A retrospective study was conducted at Songklanagarind hospital, a tertiary hospital in Thailand. This study was approved by the Institutional Review Board, Faculty of Medicine, Songklanagarind Hospital, Prince of Songkla University (IRB number REC 63-426-11-1). We retrieved the records of patients with femoral fractures undergoing long intramedullary nailing, by using the International Classification of Diseases 9 Procedure (ICD-9): code 79.15, indicating a closed reduction of fractures with internal fixation of the femur from the Hospital Information System database; from June 1, 2006, to June 30, 2020. Patients aged 20–65 years and diagnosed with femoral shaft fractures were included. Those who had a pathological fracture from a primary bone tumor, metabolic bone disease, or inadequate/missing information were excluded. The sample size was calculated based on the rates of adverse events in patients with pathological and non-pathological fractures of 23 and 7 %, respectively. According to a 95 % confidence interval, type II error of 20 %, and the ratio of 1:4; at least 50 and 200 patients with pathological and non-pathological fractures were required.

### Exposure and control

Patients who had pathological fractures of the femoral shaft using the ICD-10 code M84.4x were assigned to be the pathological group as the experimental group. Those who had traumatic femoral shaft fractures using the ICD-10 code S72.x and were assigned to be the non-pathological control group.

### Outcome measurement and independent variables

Primary outcomes measured in our study were inpatient postoperative adverse events, which were classified as surgical and medical adverse events as well as reoperation and readmission. Surgical adverse events were defined as surgical site infection that required debridement or prolonged antibiotics, wound dehiscence requiring surgical intervention, hematoma, iatrogenic nerve or arterial injury, or acute anemia necessitating blood transfusions within one day postoperatively. Medical adverse events were defined as sepsis or septic shock, unplanned intubation postoperatively, acute renal failure, pneumonia, urinary tract infection, cerebrovascular disorder, myocardial infarction, venous thromboembolism, or gastrointestinal bleeding. For secondary outcomes, the consequences of their surgical procedure one year after discharge were observed. Readmission due to the progression of their underlying metastatic cancer or problems resulting from any adverse event was recorded. Reoperation was defined as an operation performed on a previously operated femur, these was also recorded.

Patients’ characteristics, including age, gender, body mass index (BMI), and comorbidities (hypertension, diabetes mellitus, thyroid disease, heart disease, lungs disease, renal disease, liver disease, cerebrovascular disease, rheumatic disorders, hematologic disorders, osteoporosis, and immunodeficiency syndrome) were reviewed and recorded in the data recording form. Preoperative data (duration before surgery, preoperative hematocrit, and platelet), intraoperative data (total blood loss, intraoperative blood transfusion, operative time), and postoperative data (volume of drainage, duration of admission) were also recorded.

### Analysis

The data were entered in EpiData version 3.1 and analyzed using the R software version 4.0.3 (The R Foundation for Statistical Computing, 2020, Vienna, Austria). Patients’ characteristics and surgical information between the exposure and control groups were analyzed using unpaired t-test, Wilcoxon rank-sum test, or Chi-square test as appropriate. We compared adverse events, reoperation, and readmission using univariate analysis and multiple logistic regression. Potential variables associated with adverse events, with a p-value less than 0.2 on univariate analysis, were included in the multiple logistic regression models for each outcome using backward stepwise selection. The associations of exposure to all outcomes were measured by the adjusted odds ratios (adjusted OR), with a 95 % confidence interval (CI). Statistical significance was considered as a p-value less than 0.05.

## Results

**Fig. 1:**
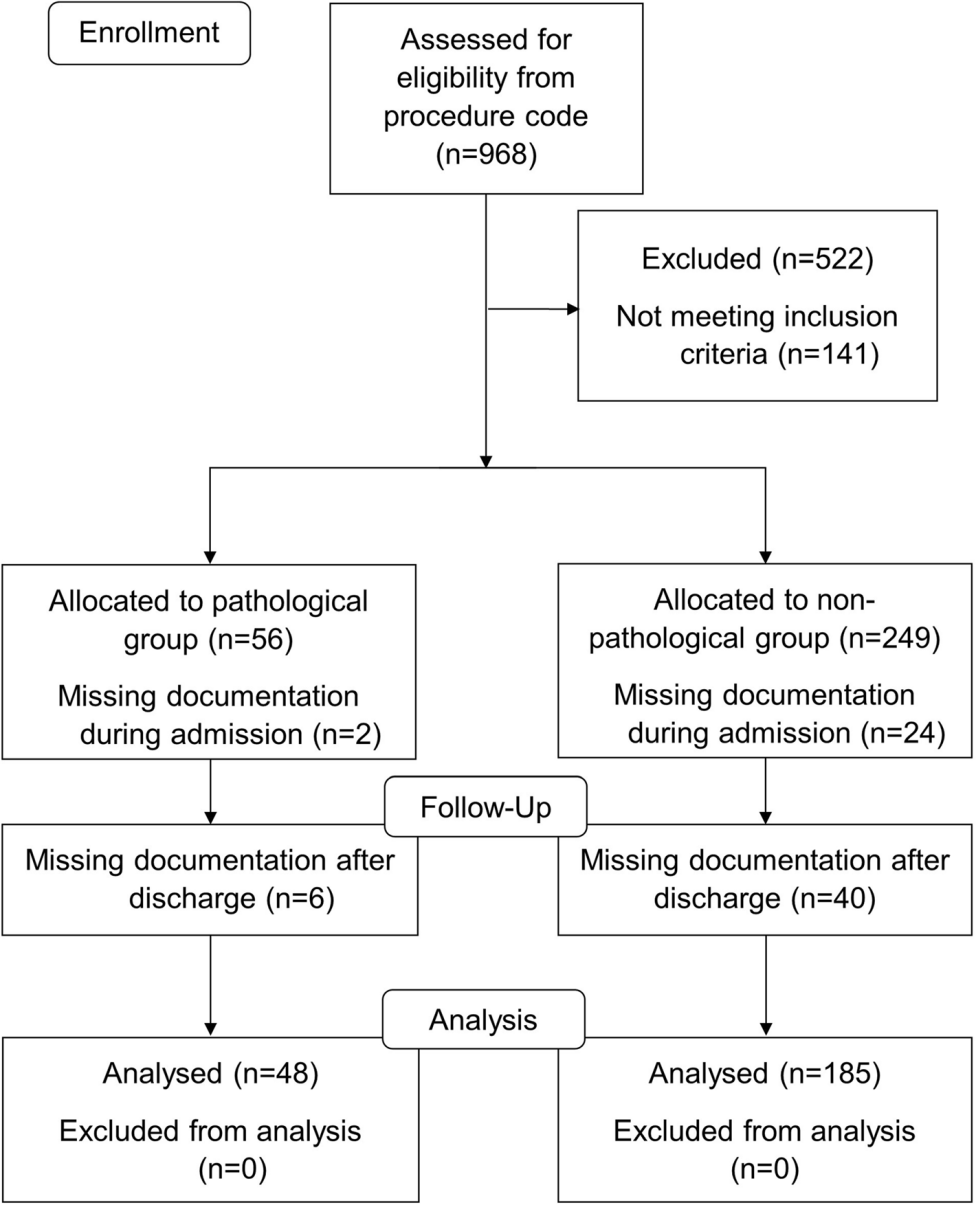
Patient enrollment flow chart

There were 56 and 249 patients, respectively, who underwent intramedullary nailing for pathological and non-pathological femoral shaft fractures during the 14-year study period. The data of 48 patients with pathological femoral fractures and 185 patients with non-pathological fractures were analyzed for adverse events. Figure 1 shows the flow chart of patient inclusion in this retrospective study. Patient characteristics compared between the two groups are shown in Table 1. Patients with pathological femoral fractures from metastatic bone disease were more likely to be female, had no comorbidity, and were fractured at the proximal location. The mechanism of injury was significantly different when compared between the two groups (p < 0.001). Table 2 shows the number of femoral fractures according to the AO Foundation/Orthopaedic Trauma Association (AO/OTA) classification system. The majority of fracture type included in this study was simple oblique fracture (AO/OTA 32-A2); however, some pathological fractures could not be classified as to the AO/OTA system due to severe osteolytic cortical bone destruction. Primary cancers that caused pathological fractures were the breast (33.3 %) and lung (33.3 %); which were predominately frequent, followed by thyroid (6.25 %), kidney (6.25 %), prostate (6.25 %), and the others were nasopharynx, esophagus, liver, or colon. Table 3 shows preoperative, intraoperative, and postoperative parameters. Preoperative hematocrit in patients with pathological fractures were significantly lower than in those with non-pathological fractures (*p* = 0.031). Duration of operative time of fewer than 325 min (p < 0.001), and volume of drainage (p = 0.003) in patients with pathological fractures were significantly higher than those with non-pathological fractures.

**Table 1:** Patient characteristics compared between the two groups

	Type of Fracture		
	Non-pathological fracture (*n* = 185), number (%)	Pathological fracture (*n* = 48), number (%)	*p* value
Age (years)*			< 0.001
Median (IQR)	28 (23,45)	60.5 (49,67.2)	
Gender*			< 0.001
Male	142 (76.8)	19 (39.6)	
Female	43 (23.2)	29 (60.4)	
Body mass index (kg/m^2^)*			0.003
Median (IQR)	22.6 (20.4,25.7)	20.1 (19.1,21.8)	
Comorbidities			0.128
At least 1 comorbidity	50 (27)	19 (39.6)	
No comorbidity	135 (73)	29 (60.4)	
Location of fracture*			< 0.001
Proximal shaft	48 (25.9)	31 (64.6)	
Midshaft	128 (69.2)	13 (27.1)	
Distal shaft	9 (4.9)	4 (8.3)	
Mechanism of injury*			< 0.001
High injury	165 (89.2)	1 (2.1)	
Low injury	20 (10.8)	13 (27.1)	
No injury	0	34 (70.8)	

*IQR* interquartile range

*Statistical significance at *p* < 0.05

**Table 2:** Femoral shaft fractures based on the AO/OTA classification system

	Femoral shaft fractures, number (%)
Simple fracture (32-A)	
Spiral fracture (32-A1)	0
Oblique fracture (32-A2)	92 (39.4)
Transverse fracture (32-A3)	58 (24.9)
Wedge fracture (32-B)	
Intact wedge fracture (32-B2)	8 (3.4)
Fragmentary wedge fracture (32-B3)	12 (5.2)
Multifragmentary fracture (32-C)	
Intact segmental fracture (32-C2)	9 (3.9)
Fragmentary segmental fracture (32-C3)	12 (5.2)
Non applicable	42 (18)

*AO/OTA* AO Foundation/Orthopaedic Trauma Association

**Table 3:** Preoperative, intraoperative, and postoperative parameters compared between the two groups

	Type of Fracture		
	Non-pathological fracture	Pathological fracture	*p* value
Preoperative parameters			
Duration before surgery (days)			0.52
Median (IQR)	8 (6,11)	8 (5,15.5)	
Preoperative Hematocrit (%)*			0.031
Median (IQR)	33.3 (30.1,38.2)	32.3 (28.7,34.4)	
Preoperative Platelet (10^9^/L)			0.131
Median (IQR)	270 (202,366)	311 (250.5,382)	
Intraoperative parameters			
Operative time (minutes)*			< 0.001
Median (IQR)	355 (267,435)	247.5 (195,310)	
Total blood loss (ml)			0.468
Median (IQR)	350 (200,500)	400 (200,725)	
Blood transfusion (ml)			0.06
Median (IQR)	0 (0,198)	0 (0,249)	
Postoperative parameters			
Volume of drainage (ml)*			0.003
Median (IQR)	80 (30,140)	120 (51.5,250)	
Duration of admission (days)			0.875
Median (IQR)	16 (12,21)	15.5 (11.8,22.8)	

*IQR* interquartile range

*Statistical significance at *p* < 0.05

**Fig. 2:**
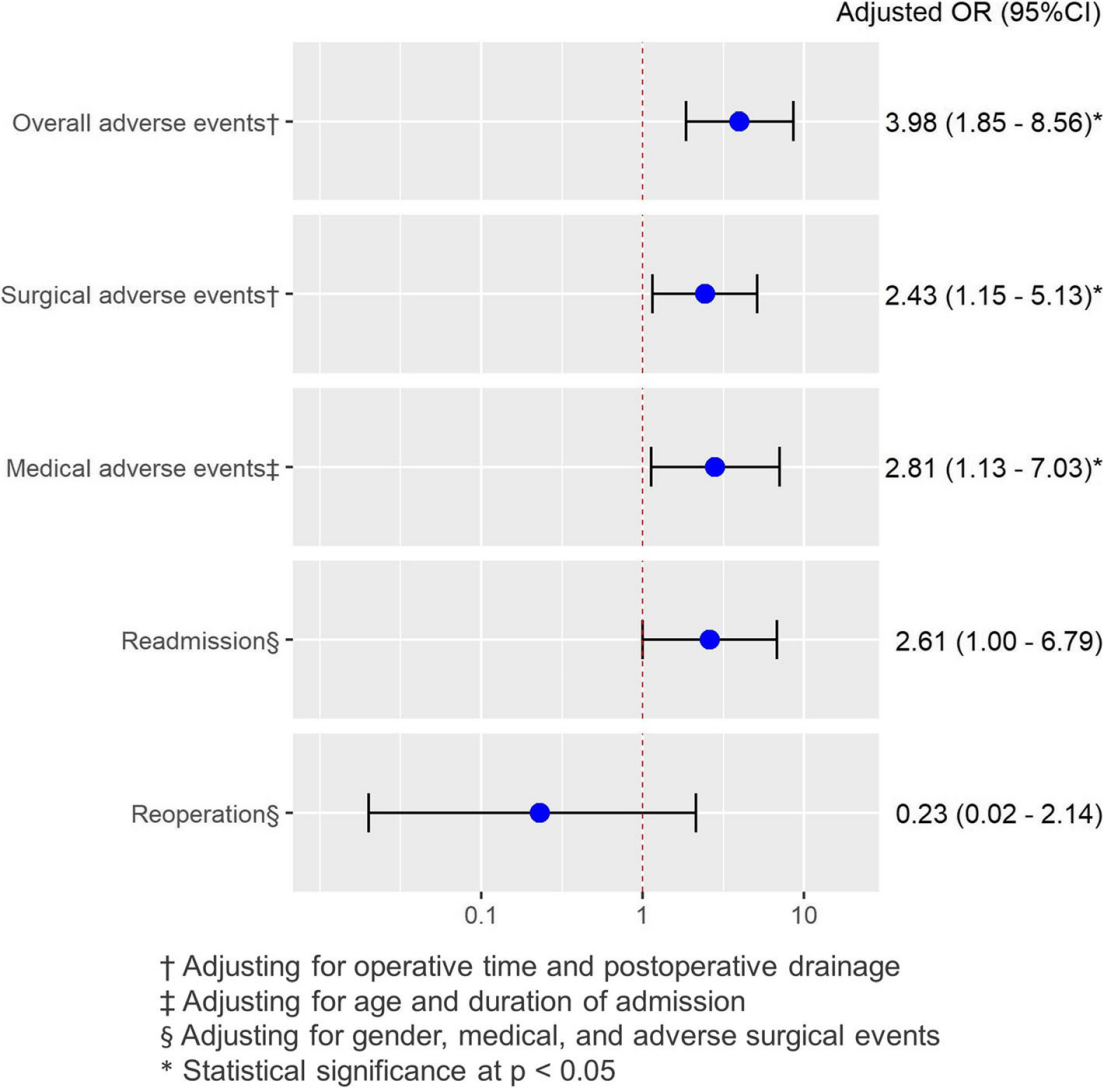
Postoperative adverse events, reoperation, and readmission in pathological fracturescompared to non-pathological fractures

The rate of adverse events was 43.7 % (102/233), of which 66.7 % (n = 32) were in the pathological fracture group and 37.8 % (*n* = 70) were in the non-pathological fracture group. The rates of surgical and medical adverse events in both groups were 36.9 % (n = 86) and 13.7 % (n = 32), respectively (Table 4). There were significantly higher rates of surgical and medical adverse events in patients with pathological fractures compared with patients with non-pathological fractures (33 % versus 52.1 % and 8.1 % versus 35.4 %). Acute anemia was the most common adverse surgical event accounting for 47.9 % in pathological fractures and 29.2 % in non-pathological fractures. Septic shock and urinary tract infections in patients with pathological fractures were more common than in the other group. However, there was no incidence of vascular injury, wound dehiscence, or cerebrovascular disorder in either group. Factors associated with adverse events in the final model of multivariate regression analysis are presented in Fig. 2. After adjusting for all potential confounding factors in multivariate regression analysis, patients with pathological fractures had higher odds of overall adverse events (adjusted OR 3.98, 95 % CI 1.85–8.56), adverse surgical events (adjusted OR 2.43, 95 % CI 1.15–5.13), and adverse medical events (adjusted OR 2.81, 95 % CI 1.13–7.03) than in those with the non-pathological fractures. In regard to consequences after discharge, there was an increased risk of readmission in the pathological group (adjusted OR 2.61, 95 % CI 1.00–6.79). On the other hand, the risk of reoperation was not different (adjusted OR 0.23, 95 % CI 0.02–2.14).

**Table 4:** Surgical and medical adverse events compared between the two groups

	Type of Fracture					
	Non-pathological fracture, number (%)		Pathological fracture, number (%)	Total	*p* value	
Surgical adverse event		61 (33)	25 (52.1)	86 (36.9)		0.023*
Surgical site infection		1 (0.5)	1 (2.1)			0.370
Hematoma		4 (2.2)	2 (4.2)			0.606
Acute anemia		54 (29.2)	23 (47.9)			0.022*
Nerve injury		6 (3.2)	0			0.350
Medical adverse event		15 (8.1)	17 (35.4)	32 (13.7)		< 0.001*
Septic shock		3 (1.6)	4 (8.3)			0.030*
Acute renal failure		3 (1.6)	2 (4.2)			0.274
Pneumonia		4 (2.2)	4 (8.3)			0.059
Urinary tract infection		2 (1.1)	4 (8.3)			0.017*
Myocardial infarction		0	1 (2.1)			0.206
Gastrointestinal bleeding		1 (0.5)	1 (2.1)			0.370
Unplanned intubation		2 (1.1)	1 (2.1)			0.501
Venous thromboembolism		3 (1.6)	3 (6.2)			0.104

*Statistical significance at *p* < 0.05

## Discussion

Rates of adverse events after surgical fixation in patients with metastatic pathological femoral fractures were greater than those with non-pathological femoral fractures after adjusting for other confounding factors. The risk of adverse surgical and medical events in the pathological group was two-fold to three-fold higher than the non-pathological group. Patients with pathological fractures were more likely to be readmitted, but less likely to be re-operated on.

Rates of overall adverse events, regardless of the fracture type, in our study were markedly high compared to findings in previous studies, which had different fracture locations and outcome measurements [3, 7, 27, 29]. Regarding outcome measurements, other studies did not define acute postoperative anemia as an adverse event, whereas this was the most common adverse event in our study, leading to higher overall adverse event rates [3, 27]. However, if anemia was dismissed, the overall adverse event rates were similar to the previous study [29]. As to fracture locations, Behnke et al. [7] also included fractures at the spine, upper, or lower extremities, not only femur fractures, as in our study. Due to the wide range of fracture locations, the rates of adverse events were probably attenuated.

Acute postoperative anemia was the most frequent adverse surgical event following intramedullary nailing, which was consistent with a previous study that assumed that performing intramedullary nailing was susceptible to bleeding during canal reaming. This resulted in postoperative anemia, which in turn required postoperative blood transfusions [30]. Moreover, significantly higher acute anemia rates have been found in patients with pathological fractures. The explanation is that a high amount of blood loss from passing the intramedullary nail through the hypervascular metastatic femoral lesion, particularly spread from thyroid, prostate, and renal cancer, may prevail the ongoing bleeding with coagulopathic state from high-energy trauma in the non-pathological group [31–33]. Although intraoperative total blood loss and blood transfusions in our study were not different between both groups, the blood loss measured by the volume of drainage was higher in the pathological group, which supported the results of postoperative anemia. No established difference in the incidence of surgical site infection, hematoma, or nerve injuries were found between both groups.

In line with adverse surgical events, risks of adverse medical events were greater in the pathological group. Septic shock and urinary tract infection were common adverse medical events that were higher in the pathological group than that of the non-pathological group. Our results are inconsistent with the findings of a previous study that included impending fractures undergoing prophylactic stabilization as a control group [27], while our study selected non-pathological fractures. High infection rates in pathological fractures could be explained through old age, low immunity, and the poor baseline status of patients with metastatic bone disease [34]. In addition, patients with pathological fractures required more time for ambulation, which increased the risk of urinary tract infection [35]. Even with slowly progressed ambulation, the incidences of venous thromboembolism events were not significantly increased in patients with pathological fractures because of medical and mechanical prophylaxis given during admission.

In our study, the patients in the pathological group had a readmission rate of more than twice the non-pathological group, which was different from a previous study measuring readmission at only 30 postoperative days [27]. Due to the longer period of data collection in our study, it tended to include more readmission events from adverse events, added with disease progression in the pathological group. Although the previous study did not report on reoperation rates, we attempted to explore this issue, and found similar reoperation rates between the pathological and non-pathological groups. These findings may result from the hypothesis that the pathological group had lower survival rates, particularly in patients experiencing postoperative complications [15, 27]. Additionally, the surgery goal for pathological fractures was only to improve quality of life; therefore, a second operation was rarely required, with the exception for postoperative complications [14, 16]. However, the goal of surgical fixation in both traumatic or non-pathological fractures is to provide stability and restore length, alignment, and rotation of the femur to achieve proper bony union. Some patients with this type of fracture were exposed to delayed union or nonunion risk factors; including, smoking history, open fractures, and severe enveloped soft tissue injury [36]. Consequently, they experienced a second surgery to assist in bone healing or to correct malalignment of the femur [37].

To our knowledge, no study has compared adverse events between patients with pathological and non-pathological femoral shaft fractures following surgical fixation. This study does have some limitations. First, this was a single-center study, which may limit the generalizability of its findings. Second, the sample size calculation considered the rates of any adverse events between the two groups that may have led to a small sample for specific adverse events. Third, we did not calculate the sample size based on multivariate analysis to identify other associated factors. Finally, this was a retrospective study in which some important data were not recorded.

Recognizing the probability of postoperative adverse events in patients undergoing intramedullary nailing will assist clinicians in providing pertinent information to both patients and their families. Moreover, as a consequence of high adverse surgical and medical events rates, preoperative patient preparation should be heeded, especially reserved blood components. During the postoperative period, hematocrit and vital sign monitoring are suggested, due to the high risks of postoperative anemia and infection. A larger multi-center, prospective study with large sample size is suggested.

## Conclusions

The risks of inpatient postoperative adverse events, especially postoperative anemia, were significantly greater in patients with metastatic pathological femoral shaft fractures than patients with non-pathological fractures undergoing intramedullary nailing after adjusting for potential confounders. Exploring through one-year follow-up, the risks of readmission in patients with pathological fractures seem higher; however, reoperation rates were not different. Counseling patients about the risk of adverse events along with well-prepared preoperatively patient care should be undertaken.

## Data Availability

Not applicable.

## Abbreviations

ICD: International Classification of Diseases
BMI: Body mass index
OR: Odds ratio
CI: Confidence interval
IQR: Interquartile range
AO/OTA: AO Foundation/Orthopaedic Trauma Association
